# Virtual patient simulation: what do students make of it? A focus group study

**DOI:** 10.1186/1472-6920-10-91

**Published:** 2010-12-04

**Authors:** Mihaela Botezatu, Håkan Hult, Uno G Fors

**Affiliations:** 1 Department of Learning, Informatics, Management and Ethics, Karolinska Institutet, Berzelius väg 3, S. 171 77 Stockholm, Sweden

## Abstract

**Background:**

The learners' perspectives on Virtual Patient Simulation systems (VPS) are quintessential to their successful development and implementation. Focus group interviews were conducted in order to explore the opinions of medical students on the educational use of a VPS, the Web-based Simulation of Patients application (Web-SP).

**Methods:**

Two focus group interviews-each with 8 undergraduate students who had used Web-SP cases for learning and/or assessment as part of their Internal Medicine curriculum in 2007-were performed at the Faculty of Medicine of Universidad el Bosque (Bogota), in January 2008. The interviews were conducted in Spanish, transcribed by the main researcher and translated into English. The resulting transcripts were independently coded by two authors, who also performed the content analysis. Each coder analyzed the data separately, arriving to categories and themes, whose final form was reached after a consensus discussion.

**Results:**

Eighteen categories were identified and clustered into five main themes: learning, teaching, assessment, authenticity and implementation. In agreement with the literature, clinical reasoning development is envisaged by students to be the main scope of VPS use; transferable skills, retention enhancement and the importance of making mistakes are other categories circumscribed to this theme. VPS should enjoy a broad use across clinical specialties and support learning of topics not seen during clinical rotations; they are thought to have a regulatory effect at individual level, helping the students to plan their learning. The participants believe that assessment with VPS should be relevant for their future clinical practice; it is deemed to be qualitatively different from regular exams and to increase student motivation. The VPS design and content, the localization of the socio-cultural context, the realism of the cases, as well as the presence and quality of feedback are intrinsic features contributing to VPS authenticity.

**Conclusions:**

Five main themes were found to be associated with successful VPS use in medical curriculum: Learning, Teaching, Assessment, Authenticity and Implementation. Medical students perceive Virtual Patients as important learning and assessment tools, fostering clinical reasoning, in preparation for the future clinical practice as young doctors. However, a number of issues regarding VPS design, authenticity and implementation need to be fulfilled, in order to reach the potential educational goals of such applications.

## Background

Virtual Patient Simulation (VPS) is a growing research field, as these software applications are considered to have entered "the mainstream of medical education" [1]. The overwhelming majority of articles published are quantitative in nature and in most cases address the use of virtual patients for learning, while only a fraction are dedicated to assessment and even fewer to other topics of interest. In spite of promising research results in the recent years and of some attempts to integrate VPS in different curricula, the "effective use requires evidence to guide design and integration" [2]. One important factor in terms of success or failure of curricular implementation of VPS is the opinion of users, both students and faculty staff. For example, if the users' expectations do not match the curricular use envisaged by the faculty or the options offered by the application itself, the possibility of a successful implementation of VPS in a medical curriculum is limited.

One way to generate the much needed information on how to best implement VPS is by means of qualitative research studies, where the different stakeholders-students, clinical teachers, course directors, faculty board-can convey their views on the role of VPS in healthcare education. Qualitative methods are extensively used in health sciences education research, especially in nursing and psychology; however, to our knowledge, qualitative methodology is not so frequently used as quantitative methodology for research on virtual patient simulation systems, with few exceptions [3-5]. Amongst qualitative methods, different interviewing techniques help researchers to detail the stakeholders' personal views [6,7].

This study aimed to report on the experiences of medical students with a virtual patient simulation system integrated in the Internal Medicine curriculum at Universidad el Bosque (Colombia). We explored the participants' opinions on the educational use of the application by using focus group interviews, as we believe that the learners' perspectives on such systems are quintessential to a successful VPS development and implementation.

## Methods

We conducted focus group interviews to explore medical students' opinions on the educational use of the system Web-based Simulation of Patients (Web-SP). The interviews were performed at the Faculty of Medicine of Universidad el Bosque (Bogota), in January 2008.

### Study design

#### The Virtual Patient application

Web-SP is an explorative linear-interactive virtual patient simulation developed at the Department of Learning, Informatics, Management and Ethics, Karolinska Institutet, Sweden [8]. A Spanish variant of the software has been developed, localized [9] and used from mid 2005 for learning and performance-based assessment in the Internal Medicine course at the Faculty of Medicine, Universidad el Bosque [10,11]. The virtual cases used at Universidad el Bosque were created from actual clinical records of patients from university hospitals in Bogota. As a consequence, the virtual cases contained patient photographs and diagnostic media pertaining to the clinical records. All patients had previously signed an informed consent form, enabling the clinical teachers to use all the information in the records as a base for case creation. In order to solve a virtual patient case, the students were required to gather information from patient interviews, physical exam and ancillary tests, in order to formulate the diagnosis and the treatment course. After submitting the treatment, the students gained access to the feedback module; in the case of the Spanish version, the feedback consisted of a detailed case discussion provided by a senior clinical expert and the actual patient follow-up.

#### Participant selection

The participants were medical students who had used Web-SP cases for learning and/or for assessment as part of their Internal Medicine curriculum in 2007. They belonged to a cohort of 216 students participating in a larger study on virtual patient assessment results [10,11]. At the time of the interviews, 49 students had their clinical rotations in Bogota and were geographically available (convenience sampling); out of the 49, 16 students were selected by simple randomization to participate in the focus-group interviews. Randomization was performed to ensure representativeness, so that students with "good experiences" with Web-SP in terms of assessment results (who could have been more positive to the educational use of the application) were not purposively selected. We deliberately recruited more students than needed, in order to allow students to decline participation or drop out at a later time. Equal numbers were recruited from the ¨study¨ and ¨control¨ arms of the study mentioned earlier, to avoid any biased opinions about the educational tool. The randomly chosen students were directly contacted by the vice dean's office and invited to participate in the study. None of the students declined participation, nor dropped out of the study. When all students confirmed their participation, we had to arrange two focus groups, as 16 participants would have been too many to accommodate in one session.

The main researcher had been the course director for Internal Medicine, implemented Web-SP in the study plan and conducted two other previous studies in the same population, so a trustful relationship between the principal investigator and the students had already been established prior to study commencement; however, the existence of a relative position of power is hereby acknowledged. Ethical permit to perform the interviews was given by the Faculty Board.

#### Setting

The interviews were conducted in facilities of the Faculty of Medicine. MB led the focus group interviews, assisted by one of the local clinical teachers. The discussion began by recapitulating their experience with the system and by stating the scope of the interviews, i.e. the exploration of their perception of the system. Then several topics were introduced, as the students had used the system for learning and/or for assessment and had also collected clinical cases in the hospitals. During the interviews the students themselves raised issues the researchers did not plan to bring up, such as communication skills development or their motivation in using the system.

#### Data collection

The interviews were conducted in Spanish, by the main investigator (MB). An interview guide had previously been developed and peer validated on site. Both audio and video recordings were made during the interviews, which were 60 and 62 minutes long, respectively. No field notes were taken during or after the focus group interviews. Each topic was discussed till estimated saturation.

### Data analysis

The audio tapes were transcribed by the main author. A non-verbatim approach was used, meaning that the transcriptions were edited for pauses, interjections and other audio utterances whose omission would not alter in any way the message conveyed by the participants. The video recordings were used to identify the speakers on the audiotapes, which were assigned consecutive numbers. The transcripts were translated into English by an authorised translator not affiliated to Karolinska Institutet or to Universidad el Bosque. The theoretical framework used in this study was content analysis [6]. MB and UF reviewed the transcripts independently, compared notes and reconciled the differences. Emerging coding was used to obtain categories and themes, whose final form was reached after another consensus discussion between the two coders. Given the manageable length of the two focus group discussions, no software was used to analyze the data.

## Results

Emerging coding identified eighteen categories in the transcripts, which were further clustered into five themes. The presentation of the results includes quotations, which are given in brackets and are followed by the student identification number. The most articulate quotation was chosen in each case, in order to avoid redundancy. However, this does not mean that other students did not express the same idea in other words and did not merely agree. When several participants concurred, the identification numbers were omitted.

For a synopsis of the results, please consult Table 1.

**Table 1:** Synopsis of themes and categories

Theme	Category	Main results
Learning	Clinical reasoning	-Clinical reasoning development is linked to a stepwise approach to case solving. -Input on factual and core knowledge is welcome as part of the VP "package", especially as feedback. -Holistic view of the patient and closure sense.

	Transferable skills	-Directly to the real patients, especially when the cases used in teaching were created from real life clinical records. -To other types of exam.
	Retention	Learning with VP enhances retention.
	Mistakes	-Recognizing and correcting mistakes in a safe environment is crucial for successful learning. - VP mistakes are less prone to be repeated in clinical practice.

Teaching	Clinical specialties	-VPS should be used in all major clinical specialties. -Topics: frequent diseases and their complications; topics not included in the study plan and in the clinical rotations; diseases that might be easily missed during a short clinical rotation (due to seasonality or to being endemic in a different geographic area).
	Regulatory effect	-Institutional level: instruction becomes uniform across rotations sites. -Individual level: limiting the availability of the system externally regulates learning.

Assessment	Qualitatively different	-...and intrinsically better evaluation tool. -VPS should not be the only assessment form used in a course. Implicit: VPS should be used for course assessment only. -Allow increased retrieval of information in comparison with regular examinations.
	Motivation	VPS can increase motivation for learning.
	Professional focus	-Assessment and feedback on assessment are perceived as important learning tools. -VP assessment should be relevant for future clinical practice as a general practitioner.
	Production assessment	-Open questions make students think. -Students favour open questions even if the grades lower.

Authenticity	Design and content	-Should reflect the real clinical practice and offer localized menu/content choices. -Might consider including actual costs. -Artificial menus/content options are misleading.
	Localization of the socio-cultural context	Necessary for applications developed in one country and implemented in another.
	Realism and virtuality	-Real life records thought to make better patient cases than fictitious scenarios. -The knowledge derived from them is directly transferable to real patients. -Strong emphasis on patient photo. -Cases created from real life patient records no longer perceived as "virtual".
	Feedback	Actual patient evolution and effect of treatment are highly desirable features of feedback, adding to realism.

Implementation	Number of cases	More than one case per topic can be necessary for common diseases which are often complicated/have co-morbidities at presentation. Min. 5-6 cases of tropical diseases.
	Access and availability	The availability of the application should be restricted in time.
	VP exchange	Tropical diseases cases should be exchanged with other HEIs.

### Theme 1: Learning

#### -Category 1: Clinical reasoning

The main educational scope of virtual patients found in the interviews is the use for training clinical reasoning-"*I believe that clinical reasoning abilities are reinforced with virtual patients*" (student 1)-, for which the constant use of VPS is deemed essential. Training with VPS is also thought to enhance analytic and synthetic reasoning-"*Analysis and synthesis...are basically the aspects that are strengthened through virtual cases...and may be reinforced by constant practice using the program*" (student 3). Clinical reasoning development is also linked to a stepwise approach to case solving, fostered by VPS-"*How I see it is that it does help the mental process of how to approach a patient, from the reason why a patient needs a consultation until the final diagnosis. When a real patient comes, we already know what to ask, what we have to do, in what order to do it, so we do not miss anything, from the reason for the consultation till diagnosis and treatment in an adequate manner without forgetting any important aspect*" (student 4). Even though clinical reasoning development is seen as the primary scope of learning with VPS, input on factual and core knowledge is welcome as part of the "package", especially as feedback-"*When receiving feedback, we learn more about pathophysiology, molecular biology etc. and thus complement the clinical part with a more theoretical, abstract part*" (student 1); the VPS scope-"*would be to reinforce clinical reasoning abilities, but also give feedback with regards to the theoretical aspects of the disease in question*" (student 8). The participants greatly appreciate that VPS enable them to follow up a patient from the beginning to the end; the cases give students a sense of closure, which enhances motivation-"*In the hospital you meet the patient in an initial phase, during the investigation or treatment, but you never know what happened later" *(student 2). The students understand VPS as a "preparation for the real life as doctors".

#### -Category 2: Transferable skills

The real cases, seen later on as a young doctor, could be solved by association and comparison with virtual patient cases with similar characteristics-"*It could happen that we remember a virtual case in order to use that experience or some of it in reality*" (student 2). The knowledge acquired with VPS is considered transferable to other types of exams. Moreover, most students consider that knowledge transfers directly to the real patients, especially when the cases used in teaching were created from real life clinical records-"*If we meet a patient with cardiac failure on Web-SP... and we meet a patient with similar characteristics in real life, then one can say: the patient has cardiac failure or possible cardiac failure and then the second part would be like choosing the indicated options through Web-SP for ordering lab tests in order to reach a diagnosis and treat the patient*" (student 6).

#### -Category 3: Retention enhancement

Students appreciate that VPS help retain information:-"*I remember more Mr. X's case than the page in the textbook*" (student 10);-"*We associate a disease more to a patient than to the textbook. If I saw the patient, saw the photo and questioned the patient in the program, I will remember more easily, I'll have my flashback of that pathology, more than if I only studied my class notes or a book*" (student 4).

#### -Category 4: Making mistakes and learning from them

Recognizing and correcting mistakes, either in the clinical reasoning path or in previously acquired knowledge, is considered crucial for successful learning-"*Web-SP lets us correct or reinforce certain knowledge so that, later, when we meet with reality or real people or cases, we won't make, or at least make less mistakes, in order to reach a correct diagnosis or adequate treatment for the patients*" (student 2). Moreover, students even think that they are less prone to repeat the mistakes made with VPS in their clinical practice-"...*so that you can go on to the clinical practice and maybe not make those same mistakes*"-as in Web-SP (student 10). Making mistakes with VPS is also not as stressful as in the real life context-"*It seems to me that it is good in part because one learns from mistakes and one is not too stressed about making mistakes, making mistakes doesn't have severe consequences*" (student 11);-"*Better to make virtual mistakes than real ones*" (student 13).

#### Communication skills

The participants consider that the development of communication skills falls out of the scope of VPS. On one hand, VPS are ill-suited for such purpose-"*Web-SP is what connects theory and practice, because it really applies to what we experience in real life, but without communication skills*" (student 5)-and on the other, communication skills are acquired by direct contact with the patient-"*I think that those skills are directly acquired in another way*" (student 5).

### Theme 2: Teaching

#### Category 1: Clinical specialties

-The participants believe that VPS should enjoy a broad use across clinical specialties. The majority of students favour the curricular use of VPS across the main clinical specialties, such as internal medicine, surgery, obstetrics-gynecology and paediatrics.

-Within a given clinical specialty, the virtual patient cases should present frequent diseases and their complications; topics not included in the study plan and in the clinical rotations should also benefit from VPS, which could close the gap-"*If we do not have rounds in a specialty, it would be good to see virtual patients, we gain by it*" (student 4). Diseases that might easily be missed during a short clinical rotation (due to seasonality or to being endemic in a different geographic area), but are relevant for the future clinical practice, should also be presented as VPS-"*Knowing about a particular disease in a specific geographic area is very important*" and "*Here in Bogota there's hardly any malaria or Leishmaniasis or yellow fever..*." (student 11). The students point out that 5-6 cases of tropical diseases would make an acceptable minimum in the study plan.

#### Category 2: Regulatory effect of VPS

A possible regulatory effect of VPS is envisaged by the participants. Different rotation locations in Colombia offer a varying infectious disease spectrum, as well as patients with different socio-economic status. VPS are seen by most students as a chance to make instruction uniform among rotations sites.

The majority of students in our study seem to require external regulation, by means of limiting the availability of the system-"*Without a time limit we can say: I'll check the cases later, and then nothing happens; but if there's a time limit, well, this week I see cardiac failure patients etc. It's more practical for us and also for the teachers, I think*" (student 5). Time restrictions help students to plan and organize their workload. The use of a system permanently left open is estimated by the participants at a surprisingly low 3%.

### Theme 3: Assessment

#### -Category 1: VPS assessment-qualitatively different from regular exams

According to the interviewees, VPS evaluate knowledge in a different manner, emphasizing the clinical reasoning process. The students consider VPS to be a more didactic form of evaluation and an intrinsically better evaluation tool than traditional exams-"*VPS evaluation lets you see your strengths and weaknesses, where you are failing and what you need to improve, while on a paper exam one can, many times, get it right just by chance*" (student 15);-"*VPS are a more didactic evaluation method because...little by little we move forward to the big question, the diagnosis and treatment of this patient, after looking at all the patient's data, including images, audio and video*" (student 1). However, students agree that VPS should not be the only assessment form used in a course. As pointed out earlier, VPS also allow an increased retrieval of information in comparison with regular examinations and therefore grades tend to be higher with virtual patient evaluation-"...*one has better chances to get information about the patient, study the case*" (student 10).

#### -Category 2: Increased motivation

VPS evaluation-"*does not feel as exam in the long run*" (student 1). For example, Web-SP features open questions, which are "harder" than closed/multiple-choice questions; despite that, they "feel more natural" and the evaluation is deemed by most students as less stressful than with other assessment methods.

#### -Category 3: Professional focus in assessment

Most students want to learn from summative assessment and from the feedback on assessment. Participants say they "*get more*" from VPS assessment-"...*the reasoning process is evaluated more, not just the answer, not just the diagnosis, but the explanation you submitted for the diagnosis, the tests you ordered, the questions you asked the patient*" (student 12);-"...*the analysis you made*" (student 14). However, assessment should always be relevant for the future clinical practice as a young doctor. VPS assessment, as well as evaluation in general terms, should be directed to-"*what a general practitioner should know on a subject*" (student 13).

#### -Category 4: Production assessment

The students are positive to open questions, which-"*make you think and analyze the patient*" (student 2). They seem to favour open questions, even if the grades might be lower than with other types of evaluation.

### Theme 4: Authenticity

#### -Category 1: Design and content

In the opinion of participants, VP design should reflect the real clinical practice. The option menus should offer realistic and localized choices in terms of history taking, physical exam and diagnostic tools, as well as feedback on treatment alternatives-"*Simulated cases should teach us how to act in real life, they have to be similar with real life practice, and if the computer gives me all those choices and I won't have access to them in the hospital, well, it doesn't give me much*" (student 5). Realism could encompass even the actual costs for the healthcare system-"*It would be great if the program showed how much a certain test costs and how much the patient and the health insurance company would have to pay*" (student 11). Too many unstructured options in a VPS may be misguiding; the abundance is not necessary to meet the learning objective-"...*there are options that might confound what you already know...you can't make a diagnosis because you're lost*" (student 15).

#### -Category 2: Localization of the socio-cultural context

In the case of applications "born" in one country and "adopted" in another, it is crucial to adapt the patient interview section to the socio-cultural context of the patient-"*If I ask the patient a question literally drawn from Web-SP he/she might say *"*I don't understand the question*" (student 10).

#### -Category 3: Realism and virtuality

The participants think that real life records make better cases than fictitious scenarios; moreover, such cases are thought to provide transferable skills. Media presence is essential to authenticity, and realism starts with the patient photo-"*The fact alone of being able to see a photo of the patient...gives us more than a paper case; it is different to see for myself that the patient is sad, then to read: the patient looks sad" *(student 16). A video recording is a must in certain circumstances:-"*In a paper case I can read that the patient is seizing, but in Web-SP I can see him seizing*" (student 10). The participants no longer perceive the cases created from real life patient records as "virtual"-"*the case was not virtual anymore because my patient was a real one*" (student12).

#### -Category 4: Feedback

Feedback on the actual evolution of the patient adds to the realism of the application. The feedback section should show the actual clinical evolution and the effect of treatment-"*It is very different to imagine the patient got better with antibiotic administration, than having proof-in the feedback-that the antibiotic actually worked*" (student 10).

### Theme 5: Implementation

As students seemed unanimous with regard to implementation strategies, no quotes are given in this section. A number of important issues were brought up by the students, including:

#### -Category 1: Number of cases

More cases were linked to more knowledge. More than one case per topic can be necessary for common diseases which are often complicated/have co-morbidities at presentation.

#### -Category 2: Access/availability

Broad access preferred in relation to site-campus, hospital, home -, but not in relation to timeframe, which should be restricted, as previously shown.

#### -Category 3: Virtual Patient exchange

The tropical diseases cases developed at Universidad el Bosque should be subject to exchange with other Higher Education Institutions.

## Discussion

Focus group interviews were the methodology chosen for this study. From the array of qualitative methods, both questionnaires and individual in-depth interviews would have been reasonable alternatives. After discussing the possibility of using in-depth interviews and questionnaires as opposed to focus groups, we chose the latter. The reason was that we intended to collect as much data as possible while still keeping the project feasible. We felt that while allowing the collection of sufficient, good quality data, it was also a practical way-financially and time-wise-to investigate the participants' opinions. The students have also come up with novel information, i.e. descriptions, explorations and ideas that brought up issues new to the researchers, which in our understanding might have not surfaced with individual interviews or questionnaires.

### Learning

*Clinical reasoning development *is already recognized by the literature as the main scope of VPS in education [2,4,10]. Our study adds the "factual and core knowledge" input via the virtual patient application, which was considered by the participants as a highly desirable feature of VPS.

Instead of re-experiencing the daily frustration of not knowing what happened with their patients (rotation ended, patient was moved to another floor etc.), the students conveyed the importance of getting closure by means of patient feedback.

The students indicate that the *transfer of knowledge *to the real patient is the ultimate goal of simulation technology, in agreement with the literature [12,4,11].

Medical students feel they remember more with VPS. *Retention enhancement *with VPS has previously been demonstrated in the same cohort of students, where the effect of VPS on early and late retention was studied [11].

*Making mistakes *was not expected to rise to the level of category. Free from a stressful context (patient, family, hospital staff), the students still perceive errors as serious events, but at the same time as meaningful learning opportunities, to the point that they consider their repetition as unlikely in their future clinical practice.

*Communication*. In our experience, VPS are not a proper tool for communication skills development. This opinion is shared by the VPS community at large and by the participants in our study. The latter found their motivation in the reduced interactivity of the system, which does not reflect the richness of direct interaction with the real patient.

### Teaching and assessment

The students in our study had used VPS both for learning and for assessment in their Internal Medicine course, where Web-SP was a curricular component. Not surprisingly, they do not envisage a use for VPS outside of the curricular context, e.g. as an add-on. They would like other major clinical specialties to offer the opportunity to work with VPS; especially so if certain topics are not comprised in the study plan, but are likely to surface in the first level of attention, finding in alignment with the recent accreditation requirements of the Liaison Committee on Medical Education (LCME) [1,13]. Other than that, they also express the need for solving cases of common, frequent diseases, particularly cases already complicated at debut, patients with several co-morbidities, as well as cases of drug interaction. To our surprise, the participants evoked a regulatory effect of VPS at individual level (to help program their learning) and at institutional level (to even out the differences in disease range among rotation sites).

As for summative assessment, we were not surprised to see that participants consider VPS a good evaluation tool [10,11]. To come across the conviction that VPS assessment is qualitatively different in comparison with other evaluation methods was however quite unexpected. A further benefit of a virtual patient application is that assessment may not feel as an exam, leading to an increased motivation for learning itself. The students are also aware that they can and should learn from assessment; feedback is deemed crucial for such learning through assessment to occur. They do not support, however, a VPS assessment directed to anything else than knowledge and skills essential for the clinical practice as a general practitioner, or the use of VPS outside regular course evaluation. Caution should be exerted when generalizing a benefit for learning across different systems, as the assessment formats are different (open-ended questions in Web-SP, multiple choice in other systems) and the feedback, if provided, also varies in layout.

### Authenticity

The *design *of the application should reflect the reality of clinical practice [4,14] and offer localized menus and content choices [9]. Authenticity might be increased by considering the actual costs involved in diagnosing and treating the virtual patient, but we believe that such a development should be subject to a localization demand. Layouts that feel artificial in terms of menus or content do not meet their educational goal and are considered by participants as downright misleading [14]. In contrast, knowledge derived from virtual cases created from real life clinical records is thought to be *directly transferred *to actual patients, which is a novel finding. An additional possible benefit of using real cases to create virtual patients is a further enhancement of authenticity to such level as to consider the application as a surrogate for reality (a desirable feature in the case of rare diseases, topics not seen during a clinical rotation, diseases unavailable geographically or seasonally etc.). Feedback derived from real cases greatly adds to the realism of the application.

In our experience, media use should serve an educational goal and not *become *a goal. The literature [2,4] supports our results, that authenticity can be conveyed by means as simple as the face photo, together with shots of the main findings of the physical exam. We were somewhat puzzled by the students' emphasis on the importance of the face photo; nevertheless, the Spanish version of Web-SP features photos of genuinely ill individuals, who may well wait in line for a consult at the clinic. The students responded empathetically to faces they considered familiar. We believe that more media resources are rarely needed. In our experience at Karolinska Institutet, the creation of such cases is unnecessarily expensive and their added value is minimal, if not null. Informed consent for any media use is an obvious must for cases created from actual clinical records, as well as for the content of the medical history itself.

### Implementation

More cases equate more knowledge in the students' opinion. However, they seem aware of the practical difficulties of achieving even a modest goal, such as one case per topic; here, as previously discussed, the options are wide: common diseases, pathologies not seen during the clinical rotations and/or not included in the study plan etc. Such difficulties are more apparent if the cases are to be created from real life records (mainly patient informed consent issues and hospital approval for retrieving information from clinical records).

Most participants recommend that the availability of the application be restricted to certain term timeslots, as a means of external regulation. Such a position is conflicting with the scope of a web-based application-namely round-the-clock access from any site-and might reflect special characteristics of the student population (whose identification was beyond the scope and the methodology of this study).

The exchange of virtual patient cases is not a priority for many institutions [14] and time will tell to what extent current inter-operability efforts are worth undertaking (e.g. as number of user sessions per case exchanged).

### Limitations

Based on our experience at LIME with VPS of a linear design, we believe our findings could be generalized to such systems. However, our setting was somewhat unusual, in the sense that the virtual patient cases were created from real life clinical records and included the actual clinical follow-up in the feedback section. Furthermore, the sample population-undergraduate medical students in Latin America-may well have different socio-cultural characteristics than participants in other studies (which makes appealing the option of designing VPS adapted to specific categories of learners).

## Conclusions

Our study found five main themes to be associated with successful VPS use in medical curriculum: Learning, Teaching, Assessment, Authenticity and Implementation. Medical students perceive Virtual Patients as important learning and assessment tools, fostering clinical reasoning, in preparation for the future clinical practice as young doctors. However, an application must comply with certain design, authenticity and implementation requirements, in order to reach its educational goal.

## Competing interests

The authors declare that they have no competing interests.

## Authors' contributions

MB conducted the focus group interviews. MB and UF independently coded the data. MB and UF performed the content analysis. All authors contributed substantially to the conception and design of the study, as well as to the critical revision of the paper. All authors approved the final manuscript.

## Authors' information

MB is a physician specialized in hematology and a doctoral candidate at the Department of Learning, Informatics, Management and Ethics (LIME), Karolinska Institutet. MB has several years' experience as a clinical teacher and is fluent in Spanish. HH is visiting professor in Medical Education at LIME. UF, professor of Medical Educational Simulation, is the head of LIME.

## Pre-publication history

The pre-publication history for this paper can be accessed here:

http://www.biomedcentral.com/1472-6920/10/91/prepub
